# RESTING STATE NETWORK AND MINDFULNESS IN MOTION PLUS DASH DIET IN AFRICAN AMERICANS WITH MILD COGNITIVE IMPAIRMENT

**DOI:** 10.1093/geroni/igz038.417

**Published:** 2019-11-08

**Authors:** Kathy D Wright, Klatt Maryanna, Ingrid Adams, Cady Block, Todd Monroe, Zhong-Lin Lu, Doug Scharre, Lorraine Mion

**Affiliations:** 1 The Ohio State University College of Nursing, Columbus, Ohio, United States; 2 The Ohio State University College of Medicine, Columbus, Ohio, United States; 3 The Ohio State University College of Medicine, Ohio, United Arab Emirates; 4 The Ohio State Wexner Medical Center, Columbus, Ohio, United States; 5 The Ohio State University Department of Psychology, Columbus, Ohio, United States; 6 Center for Cognitive and Memory Disorders, Center for Neuromodulation, The Ohio State University Wexner Medical Center, Columbus, Ohio, United States

## Abstract

The resting state network (RSN) is a target of interest in neurodegenerative research, with evidence linking functional connectivity of its constituent nodes with mild cognitive impairment and dementia. Given the emerging linkage between Alzheimer’s disease and related dementia disorders (ADRD) and hypertension (HTN), non-pharmacological interventions that promote RSN connectivity and blood pressure are needed. The purpose of this pilot study protocol is to deliver a novel intervention, combining mindfulness and the Dietary Approaches to Stop Hypertension (DASH), to improve RSN connectivity and blood pressure in African American (AA) older adults with MCI and HTN. Thirty-six AAs aged 65 and older will be randomized to mindfulness plus DASH, attention control (non-health related education), or a control group. The Mindfulness in Motion (MIM) plus DASH intervention is delivered in 8-weekly group sessions of 6-10 participants. MIM includes mindful movements from chair/standing, breathing exercises and guided meditation. The DASH intervention uses a critical thinking approach that involves problem solving, goal setting, reflection, and developing self-efficacy. Both components are culturally tailored for older African Americans. Cognitive examination, diet and mindfulness practice surveys, blood pressure, and functional magnetic resonance imaging (RSN) data are collected at baseline and 3 months. Forty-eight AAs were screened and 17 were enrolled (women= 13; men= 4) to date. Of the 17 enrolled, 7 were eligible for neuroimaging. Findings from this pilot study may provide the preliminary evidence that MIM plus DASH may improve RSN connectivity and blood pressure in this population at risk for ADRD.

